# Independent and Combined Associations of Physical Activity, Sedentary Time, and Activity Intensities With Perceived Stress Among University Students: Internet-Based Cross-Sectional Study

**DOI:** 10.2196/20119

**Published:** 2020-11-11

**Authors:** Shu Ling Tan, Malte Jetzke, Vera Vergeld, Carsten Müller

**Affiliations:** 1 Department for Social Sciences of Sport Institute for Sport and Exercise Science University of Münster Münster Germany; 2 Department of Sport and Exercise Psychology Institute for Sport and Exercise Science University of Münster Münster Germany; 3 University Sports University of Münster Münster Germany; 4 Department of Physical Therapy European University of Applied Sciences Cologne (Köln) Germany

**Keywords:** physical activity, sedentary time, intensity of physical activity, perceived stress, university students, internet-based cross-sectional study, independent associations, combined associations

## Abstract

**Background:**

Mental health is an emerging topic on university campuses, with students reporting higher levels of psychological distress than the general population of the same age. Increasing physical activity and reducing sedentary time have been proved promising measures to promote mental health in the general population. However, to derive and implement effective measures to promote mental health among university students, further exploration of the associations between physical activity, sedentary time, and perceived stress in this specific setting is needed.

**Objective:**

This study aims to identify associations between physical activity, sedentary time, and perceived stress after controlling for sociodemographic and behavioral variables among university students in Germany. We hypothesize that perceived stress is inversely related to physical activity and positively associated with sedentary time. Furthermore, we hypothesize that combined associations of concurrently high physical activity and low sedentary time on perceived stress are stronger compared with either alone and that the association between physical activity and perceived stress depends on activity intensity.

**Methods:**

We conducted cross-sectional analyses from a large-scale internet-based student health survey (n=4189; response rate=10.0%). Physical activity, sedentary time, and engaging in moderate and vigorous activity intensities were assessed using the International Physical Activity Questionnaire Short Form with categorization into low, intermediate, and high levels. We measured perceived stress using the 10-item Perceived Stress Scale (range 0-40).

**Results:**

The results indicate that higher physical activity and lower sedentary time are associated with reduced levels of perceived stress. Following adjustment for gender, BMI, income, fruit and vegetable intake, alcohol consumption, and sleep quality, perceived stress scores were lower for students reporting high physical activity levels and low sedentary time compared with the least active and highly sedentary students (Perceived Stress Scale –2.2, 95% CI –2.9 to –1.5, *P*<.001 for physical activity and –1.1, CI 95% –1.7 to –0.5, *P*<.001 for sedentary time). Combined associations with perceived stress revealed that students concurrently reporting high total physical activity and low sedentary time reported the lowest perceived stress scores of all possible combinations following adjustment for confounders (Perceived Stress Scale –3.5, CI 95% –4.6 to –2.5, *P*<.001 compared with students reporting low physical activity levels and concurrently high sedentary time). Associations between vigorous physical activities and perceived stress were not stronger compared with moderate activity intensities.

**Conclusions:**

Self-reported physical activity and low sedentary time are favorably associated with perceived stress, while the intensity of physical activities seems to be of minor importance. These results help to effectively implement health-promoting measures on campus among university students through increasing physical activity and reducing sedentary time.

## Introduction

### Background

Insufficient physical activity (PA) with increased sedentary time (ST) and stress are major problems among young adults. Globally, 1 in 4 adults does not meet the recommendations on PA for health [1]. Several reasons for being insufficiently physically active during the transition into adulthood and especially during university years have been proposed such as lack of time or social support; stress and tiredness attributable to study overload; and structural barriers like homework, class schedules, and overcrowded facilities [2,3]. With the increased use of technology, young adults tend to be less physically active and exhibit extensive sitting times [1,4]. Additionally, major life changes might also contribute to a decline in PA that potentially impact the level of perceived stress among students, like living away from home and adjusting to a new social environment, experiencing financial difficulties, and maintaining high levels of academic achievement [5,6].

Previous studies indicate a substantial decrease in PA during the transition from late adolescence into adulthood [7] and about half of university students in Western countries are not sufficiently active to gain health benefits [8-10]. Furthermore, according to self-reports, university students spend about 7½ hours per day being sedentary, putting them at increased risk for detrimental health outcomes [11]. This is worrying given the fact that PA habits are likely to be established in young adulthood and persist throughout life [12]. This behavioral change is of particular importance because the lack of PA is one of the top 3 modifiable risk factors of chronic disease and premature death at a later age [1,13].

### Perceived Stress Among University Students

University students, particularly female students, appear to experience greater psychological distress [14,15] and higher levels of depression and anxiety [16,17] than the general population. Recent national data from one of the main health insurance providers in Germany indicate that 25% of the university students show symptoms of burnout and report doubts regarding their chosen study program. Moreover, 15.6% report symptoms of poor mental health and depression, and 17.4% report symptoms of a generalized anxiety disorder [18].

High levels of perceived stress have consistently been attributed to academic and social stressors within the university setting, such as academic workload [19], interpersonal relationships [20], and the transition to living independently [21]. Interestingly, students engaging in multiple health risk behaviors, such as physical inactivity, smoking, and unhealthy diet, reported the poorest mental health, particularly as it relates to stress [22].

### Associations of Physical Activity, Sedentary Time, and Perceived Stress

#### Overall Physical Activity and Perceived Stress

Previous reviews analyzing evidence from cross-sectional, prospective, and experimental studies have demonstrated that PA has potential to reduce perceived stress [23,24]. Various neurobiological pathways and psychosocial factors have been discussed to theoretically explain the association of PA and mental health in general and particularly on stress. Accordingly, PA is often recommended as a strategy for managing stress in adult populations [25]. However, according to a recent systematic review, a research gap still exists regarding the association of PA with stress among university students [26]. The few studies examining the relationship between PA or exercise and mental health among university students suggest that higher PA, particularly aerobic activity, is associated with lower levels of perceived stress [27,28]. Similarly, a study examining low, intermediate, and high PA groups and associations with anxiety and depression scales found that higher activity levels were associated with better self-reported mental health [29]. In addition, studies discovered positive relationships between PA and self-rated quality of life [30] and inverse associations between PA and perceived stress [31].

#### Sedentary Time and Perceived Stress

Increased use of technology is one of the main reasons for not meeting the recommended level of PA for health [1]. Students’ sedentary behavior steadily increases due to the growing use of multiple mobile devices used for studying, staying in touch with family and friends, playing computer games, and watching television [4]. These screen-based activities are strongly associated with sedentary behavior and stress, and thus, ST has been suggested as an independent predictor of metabolic risk and mental health outcomes [32], with inflammatory responses and psychosocial mechanisms being likely pathways to explain this association [28,33,34]. A few studies examined the relationship of ST with stress among university students with contradictory findings: while high ST was associated with high levels of perceived stress [35,36], recently published device-based data indicate that only leisure-time sedentary behavior, but not total ST, was associated with perceived stress [28]. Hitherto, only one study examined combined associations between PA, ST, and perceived stress and found significant combined associations in female but not in male university students [36].

#### Activity Intensity and Perceived Stress

Apart from the total volume of PA, it has recently been proposed that the activity intensity might mediate the association of PA and mental health. Research investigating the differential effects of vigorous PA (vPA) and moderate PA (mPA) on perceived stress among university students is scarce and equivocal. For instance, Gerber et al [37] examined whether vPA provides additional mental health benefits beyond mPA. In fact, their results show that vPA was associated with decreased perceived stress, pain, subjective sleep complaints, and depressive symptoms. These findings are supported by previous studies on stress, anxiety, and depression among the general population [38,39]. Contrarily, Paolucci et al [40] demonstrated that vPA (high-intensity interval training) evoked increased levels of perceived stress, and mPA might represent an optimal activity intensity for the promotion of mental health among university students.

### Hypotheses

Although the associations between PA and stress have been investigated previously among adults and college students, studies among university students are scarce and partly contradictory. To derive and implement appropriate health promotion measures in the university setting, the associations between PA, ST, and perceived stress needs further examination. Based on current literature, we hypothesize the following:

- Perceived stress is inversely related to overall PA and positively associated with ST
- The combined association of PA and ST with perceived stress is stronger than the associations of either variable alone
- The association between PA and perceived stress depends on the intensity of PA, and this relationship is stronger for vPA compared with mPA

## Methods

### Study Design and Setting

This study adhered to the Checklist for Reporting Results of Internet E-Surveys (CHERRIES) guidelines (Multimedia Appendix 1) [41]. A cross-sectional online study was implemented at the University of Münster in Germany. Ethical approval was obtained from the university institutional review board. The health survey was completed during the summer term in year 2019. All regular university students were eligible to take part in this internet-based health survey except cross-registered students, auditing students, and senior citizen students. The remaining 42,630 students were invited by email to take part. We offered the questionnaire in German and English languages to provide international students the opportunity to participate. Students received an invitation email and were provided with an individual transaction number. The email included information on the length of the survey (20 to 30 minutes, 172 items on 15 pages), voluntariness, anonymity, data protection, and incentives (eg, a chance to win VIP tickets for sporting events). Students not replying to the invitation were reminded twice within 2 weeks to take part. Prior to the online survey, all participants gave informed consent.

The survey was administered without randomization of items using the evaluation software EvaSys version 8.0 with adaptive questioning (Electric Paper Evaluationssysteme GmbH). Students were able to change their answers by using a back button. No completeness check was available. However, incomplete surveys were captured as well.

### Measurements

#### Physical Activity

Students’ PA levels and total ST were assessed using the International Physical Activity Questionnaire Short Form (IPAQ-SF) [42]. This questionnaire included 7 questions to assess vPA, mPA, walking, and ST related to the previous week by asking for frequency (days per week) and duration (minutes per day). Valid answers require PA durations of at least 10 minutes but no more than 180 minutes in each category, allowing for calculating weekly MET (metabolic equivalent of task) minutes. Vigorous MET minutes per week are calculated by multiplying the product of frequency and duration with the factor 8. Moderate and walking MET minutes are obtained using the factors 4 and 3.3, respectively. ST was assessed with a single question about the average daily ST in hours during the previous week. Missing PA or ST data were considered as completely missing for this case and not considered for statistical analysis (n=80). The IPAQ-SF has acceptable measurement properties, as demonstrated in adult populations showing adequate criterion validity against accelerometry (Spearman ρ=.30, CI 95% .23 to .36) and acceptable test-retest reliability (ρ=.76, CI 95% .73 to .77) [42]. Furthermore, the IPAQ-SF demonstrates reasonable validity in university students with Pearson *r* ranging from .27 to .70 when compared with accelerometer counts and mPA to vPA uniaxial and triaxial cut points [43].

#### Perceived Stress

The Perceived Stress Scale (PSS) [44] is a widely used 10-item self-report scale representing a reliable, valid, and economic instrument for assessing perceived stress [45]. Students responded on a 5-point Likert scale ranging from 0 = never to 4 = very often to the degree they appraised life situations as overwhelming, unpredictable, and uncontrollable (eg, In the last month, how often have you been upset because of something that happened unexpectedly?). After recoding the positively stated items 4, 5, 7, and 8, a total score is obtained by summing all 10 items to a PSS score ranging between 0 to 40. Higher scores indicate increased levels of perceived stress, but there are no predefined cutoffs. Ipsative mean imputation was used (n=70) when not more than 1 item of the complete scale was missing. In case of 2 or more missing items, the subject was not considered for statistical analysis (n=14). In this study, internal consistency was good to excellent with Cronbach alpha=.88.

#### Potential Confounders

Several sociodemographic and behavioral factors were considered a priori as potential confounding variables. Sociodemographic confounders encompass gender (dichotomized), BMI (kg/m²), total number of semesters studied, and income categorized into 5 levels: <450 EUR, 450-699 EUR, 700-949 EUR, 950-1150 EUR, and >1150 EUR. Behavioral confounders include current smoking status (dichotomized), alcohol use, sleep quality, and fruit and vegetable intake categorized into 4 levels: no servings per day, 1 to 2 servings per day, 3 to 4 servings per day, and ≥5 servings per day (equivalent to the current nutrition recommendation). Alcohol consumption was assessed using the Alcohol Use Disorders Identification Test for Consumption (AUDIT-C), a 3-item questionnaire that is recommended as a short and valid screening for hazardous drinking among university students [46]. Sleep quality was assessed using the short-form Pittsburgh Sleep Quality Index (sPSQI), a 13-item questionnaire assessing sleep quality over a 4-week time interval that demonstrates high agreement with the original 19-item survey [47].

#### Data Reduction

PA data were analyzed according to the Guidelines for Data Processing and Analysis of the International Physical Activity Questionnaire [48], resulting in 3 levels of total PA. A high PA level requires vigorous intensity activity on at least 3 days and accumulating at least 1500 MET minutes per week or a combination of walking, mPA, and/or vPA accumulating at least 3000 MET minutes per week. The level of PA is classified as intermediate with either 3 or more days of vPA of at least 20 minutes per day, 5 or more days of mPA and/or walking of at least 30 minutes per day, or accumulating at least 600 MET minutes per week of any combination of walking, mPA, or vPA. All individuals not meeting these criteria are considered to have a low PA level. ST was categorized according to previous studies in <6 hours per day, 6 to 8 hours per day, and >8 hours per day for low, intermediate, and high levels of ST, respectively [49-51].

Average MET scores are derived for the different intensities of PA (ie, mPA = 4 METs and vPA = 8 METs). Based on these values we categorized mPA and vPA into 3 different levels based on the above mentioned classification of high PA levels: a high level of vPA requires the accumulation of ≥1500 MET minutes per week of vPA, while an intermediate level is achieved by accumulating 600 to 1499 MET minutes per week of vPA. Individuals not meeting the above criteria were categorized as having a low level of vPA. Accordingly, accumulating ≥750 MET minutes, 300 to 749 MET minutes, and <300 MET minutes of mPA resulted in high, intermediate, and low levels of mPA, respectively.

### Statistical Analysis

Descriptive statistics include mean and standard deviation. Gender differences for dependent and independent variables were analyzed using chi-square tests for ordinal data and multivariate analysis of variance with Bonferroni adjustments for continuous variables. We used generalized linear models to assess combined associations for PA by ST interaction with perceived stress and to assess the association between different activity intensity levels (ie, vPA by mPA interaction) and perceived stress using linear regression models with robust estimator covariance matrix. We categorized exposure data (PA level and ST) into 3 groups, with the first level (lowest total PA and highest ST) as reference. Combined associations between PA and perceived stress were analyzed without any adjustments (model A), with adjustments for sociodemographic variables (model B), and with additional adjustments for behavioral factors (model C). The results are presented as regression coefficient B with Wald 95% confidence intervals. Independent associations between PA variables and perceived stress are presented as unstandardized (B) and standardized regression coefficients (ß) and were analyzed using fully adjusted generalized linear regression models and pairwise comparisons with Bonferroni adjustments. The level of statistical significance was set at *P*<.05. All analyses were performed using SPSS Statistics version 26 (IBM Corp).

## Results

### Participants and Descriptive Data

In all, 4189 students participated in this online survey, resulting in an overall response rate of 10.0% (4189/42,630, range 7.2% to 22.1% among the 21 university departments). A total of 67.8% (2840/4189) were female. Participant characteristics by gender are summarized in Table 1. Results of a multivariate analysis of variance indicated significant gender differences in perceived stress and sociodemographic and behavioral variables (*F*_5, 3967_=67.58, *P*<.001, ηp²=.08). Particularly, females reported higher perceived stress (mean 20.0 [SD 6.9]) compared with male students (mean 17.6 [SD 7.2]; *F*_1, 3971_=95.72, *P*<.001, ηp²=.02). No gender differences were found for ST (χ^2^_2, N=4121_=4.70, *P*=.095). However, female students were more likely to report intermediate PA levels (1348/2795, 48.2%), whereas male students were more likely to report low (156/1325, 11.8%) and high (614/1326, 46.3%) PA levels (χ^2^_2, N=4120_=23.75, *P*<.001). Male students were more likely to report engaging in high amounts of vPA (χ^2^_2, N=4120_=16.64, *P*<.001). Gender, income, fruit and vegetable intake, alcohol consumption, sleep quality (all *P*<.001), and BMI (*P*=.02) were significant covariates and explained 23.0% of the variance in perceived stress (*F*_6, 3942_=197.10, *P*<.001).

**Table 1:** Characteristics of university students by gender.

Characteristic		Total (n=4189)	Female (n=2840)	Male (n=1349)	*P* value
Age in years, mean (SD)		23.7 (4.3)	23.4 (3.9)	24.4 (5.0)	<.001^a^
BMI, mean (SD)		22.8 (3.8)	22.3 (3.9)	23.7 (3.6)	<.001^a^
Number of semesters, mean (SD)		8.4 (5.5)	8.1 (4.9)	9.3 (6.5)	<.001^a^
**Income (EUR), n (%)**		—^b^	—	—	<.001^c^
	<450	863 (18.4)	531 (18.9)	232 (17.3)	
	450-699	1273 (30.6)	907 (32.2)	366 (27.3)	
	700-949	1120 (27.0)	776 (27.6)	344 (25.7)	
	950-1150	464 (11.2)	302 (10.7)	162 (12.1)	
	>1150	534 (12.9)	299 (10.6)	235 (17.6)	
Current smoker, n (%)		379 (9.4)	215 (7.8)	164 (12.7)	<.001^c^
**FVI^d^, n (%)**		—	—	—	<.001^c^
	0 servings per day	64 (1.6)	24 (0.9)	40 (3.1)	
	1-2 servings per day	2094 (52.0)	1269 (46.2)	825 (64.2)	
	3-4 servings per day	1552 (38.5)	1216 (44.3)	336 (26.1)	
	≥5 servings per day	319 (7.9)	235 (8.6)	319 (6.5)	
AUDIT-C^e^, mean (SD)		3.5 (2.3)	3.2 (2.1)	4.0 (2.6)	<.001^a^
sPSQI^f^, mean (SD)		4.6 (2.2)	4.7 (2.2)	4.4 (2.1)	<.001^a^
**PA^g^ class, n (%)**		—	—	—	<.001^c^
	Low	377 (9.2)	221 (7.9)	156 (11.8)	
	Intermediate	1903 (46.2)	1348 (48.2)	555 (41.9)	
	High	1840 (44.7)	1226 (43.9)	614 (46.3)	
**ST^h^ class, n (%)**		—	—	—	.095^c^
	Low	663 (16.1)	472 (16.9)	191 (14.4)	
	Intermediate	1650 (40.0)	1119 (40.0)	531 (40.0)	
	High	1808 (43.9)	1204 (43.1)	604 (45.6)	
**vPA^i^ class, n (%)**		—	—	—	<.001^c^
	Low	1735 (42.1)	1209 (43.3)	526 (39.7)	
	Intermediate	1379 (33.5)	956 (34.2)	423 (31.9)	
	High	1006 (24.4)	630 (22.5)	376 (28.4)	
**mPA^j^ class, n (%)**		—	—	—	.027^c^
	Low	1248 (30.3)	815 (29.2)	433 (32.7)	
	Intermediate	1595 (38.7)	1117 (40.0)	478 (36.1)	
	High	1277 (31.0)	863 (30.9)	414 (31.2)	
PSS^k^ score, mean (SD)		19.2 (7.1)	20.0 (6.9)	17.6 (7.2)	<.001^a^

^a^MANOVA: multivariate analysis of variance.

^b^Not applicable.

^c^Chi-square.

^d^FVI: fruit and vegetable intake.

^e^AUDIT-C: Alcohol Use Disorders Identification Test for Consumption.

^f^sPSQI: short Pittsburgh Sleep Quality Index.

^g^PA: physical activity.

^h^ST: sedentary time.

^i^vPA: vigorous physical activity.

^j^mPA: moderate physical activity.

^k^PSS: Perceived Stress Scale.

### Independent Associations of Physical Activity and Sedentary Time With Perceived Stress

Generalized linear regression models were performed to analyze independent associations between perceived stress and PA variables after adjusting for covariates (Table 2). The strongest associations with perceived stress were found for total PA. Consistently, higher PA levels (*P*<.001), lower ST (*P*<.001), and higher engagement in mPA and vPA (*P*≤.01) were associated with lower perceived stress. Highly active students report –0.7 (CI 95% –1.2 to –0.2, *P*=.003) and –2.2 (CI 95% –3.1 to –1.3, *P*<.001) lower mean perceived stress scores compared with students categorized as intermediate and low physically active, while students in the intermediate PA category report –1.5 (CI 95% –2.4 to –0.6, *P*<.001) lower mean perceived stress scores compared with students categorized as least physically active. A comparable trend with weaker associations was found for ST with the exception that low and intermediate ST groups did not differ in perceived stress scores, with a mean difference of –0.1 (CI 95% –0.8 to 0.6, *P*>.99).

**Table 2:** Independent associations of physical activity levels, sedentary time, and perceived stress^a^.

Characteristic		Perceived stress			
		Mean (95% CI)	B^b^ (95% CI)	*P* value	ß^c^ (95% CI)
**PA^d^ level**					
	Low	20.8 (20.1 to 21.5)	0 (reference)	—	1 (reference)
	Intermediate	19.2 (18.9 to 19.5)	–1.53 (–2.28 to –0.79)	<.001	0.22 (0.10 to 0.45)
	High	18.6 (18.3 to 18.9)	–2.21 (–2.96 to –1.46)	<.001	0.11 (0.05 to 0.23)
**ST^e^ level**					
	Low	20.2 (19.9 to 20.6)	0 (reference)	—	1 (reference)
	Intermediate	19.2 (18.9 to 19.6)	–0.99 (–1.41 to –0.56)	<.001	0.37 (0.25 to 0.57)
	High	19.1 (18.6 to 19.6)	–1.10 (–1.66 to –0.54)	<.001	0.33 (0.19 to 0.58)
**vPA^f^ level**					
	Low	19.6 (19.3 to 19.9)	0 (reference)	—	1 (reference)
	Intermediate	19.0 (18.7 to 19.3)	–0.62 (–1.08 to –0.17)	.007	0.54 (0.34 to 0.84)
	High	18.8 (18.4 to 19.2)	–0.86 (–1.38 to –0.34)	.001	0.42 (0.25 to 0.71)
**mPA^g^ level**					
	Low	19.7 (19.3 to 20.1)	0 (reference)	—	1 (reference)
	Intermediate	19.1 (18.8 to 19.4)	–0.61 (–1.09 to –0.14)	.011	0.54 (0.34 to 0.87)
	High	18.6 (18.2 to 18.9)	–1.11 (–1.63 to –0.60)	<.001	0.33 (0.20 to 0.55)

^a^Regression model adjusted for gender, BMI, income, fruit and vegetable intake, alcohol consumption, and sleep quality.

^b^B: unstandardized regression coefficient.

^c^ß: standardized regression coefficient.

^d^PA: physical activity.

^e^ST: sedentary time.

^f^vPA: vigorous physical activity.

^g^mPA: moderate physical activity.

Pairwise comparisons indicate that associations with perceived stress were similar for engaging in vPA and mPA when comparing the intermediate with the low physically active group (vPA –0.6 [CI 95% –1.2 to –0.1, *P*=.02], mPA –0.6 [CI 95% –1.2 to 0, *P*=.03]). The comparison of high versus intermediate and high versus low engagement in both activity intensity categories indicates a stronger association for mPA with perceived stress compared with vPA and stress (vPA high vs intermediate –0.2 [CI 95% –0.9 to 0.4, *P*>.99] and mPA high vs intermediate –0.5 [CI 95% –1.1 to 0.1, *P*=.11]; vPA high vs low –0.9 [CI 95% –1.5 to –0.2, *P*=.004] and mPA high vs low –1.1 (CI 95% –1.7 to –0.5, *P*<.001]).

### Combined Associations With Perceived Stress

Generalized linear regression models with interaction effects examined the combined association of PA by ST with perceived stress (Table 3), as well as of vPA by mPA with perceived stress (Table 4). Analyses include an unadjusted model (model A), a model adjusted for sociodemographic factors (model B), and a model additionally adjusted for behavioral factors (model C).

All models consistently indicate that higher total PA and lower ST are associated with reduced perceived stress scores. The interaction of total PA and ST also reveals that for the least active students, the transition from a high to an intermediate and from a high to a low ST is associated with mean perceived stress score reductions of –1.3 (*P*=.14) to –1.4 (*P*=.07) and –2.2 (*P*=.045) to –2.7 (*P*=.02), respectively. Contrarily, these transitions are minor for students categorized as intermediately physically active and marginal for the most active students (Table 3).

The combined analysis of vPA and mPA indicates consistent inverse associations with perceived stress (Table 4). Students engaging in high amounts of vPA and high amounts of mPA revealed the strongest associations with perceived stress with significantly lower scores ranging from –3.6 in the unadjusted model to –2.1 in the fully adjusted model (each *P*<.001) compared with students in the reference group.

**Table 3:** Combined associations of physical activity levels and sedentary time on perceived stress.

PA^a^ and ST^b^ levels		Perceived stress					
		Model A^c^ (n=4072), B (95% CI)	*P* value	Model B^d^ (n=4017), B (95% CI)	*P* value	Model C^e^ (n=3945), B (95% CI)	*P* value
**Low**							
	High	0 (reference)		0 (reference)		0 (reference)	
	Intermediate	–1.26 (–2.94 to 0.42)	.14	–1.32 (–2.97 to 0.33)	.12	–1.40 (–2.91 to 0.10)	.07
	Low	–2.46 (–4.69 to –0.22)	.03	–2.71 (–4.92 to –0.50)	.02	–2.16 (–4.27 to –0.05)	.045
**Intermediate**							
	High	–1.66 (–2.73 to –0.58)	.003	–1.95 (–3.02 to –0.88)	<.001	–1.74 (–2.74 to –0.75)	.001
	Intermediate	–3.07 (–4.14 to –1.99)	<.001	–3.39 (–4.46 to –2.32)	<.001	–2.80 (–3.80 to –1.81)	<.001
	Low	–3.53 (–4.80 to –2.26)	<.001	–3.94 (–5.19 to –2.69)	<.001	–2.78 (–3.95 to –1.62)	<.001
**High**							
	High	–2.79 (–3.89 to –1.69)	<.001	–3.02 (–4.11 to –1.93)	<.001	–2.54 (–3.55 to –1.53)	<.001
	Intermediate	–4.15 (–5.23 to –3.07)	<.001	–4.24 (–5.31 to –3.17)	<.001	–3.35 (–4.36 to –2.34)	<.001
	Low	–4.16 (–5.37 to –2.94)	<.001	–4.46 (–5.67 to –3.26)	<.001	–3.51 (–4.62 to –2.39)	<.001

^a^PA: physical activity.

^b^ST: sedentary time.

^c^Model A: unadjusted regression model.

^d^Model B: regression model adjusted for sociodemographic variables (sex, BMI, income).

^e^Model C: regression model adjusted for sociodemographic and behavioral variables (model B plus fruit and vegetable intake, alcohol use, and sleep quality).

**Table 4:** Combined associations of vigorous and moderate physical activity on perceived stress.

vPA^a^ and mPA^b^ levels		Perceived stress					
		Model A^c^ (n=4072), B (95% CI)	*P* value	Model B^d^ (n=4017), B (95% CI)	*P* value	Model C^e^ (n=3945), B (95% CI)	*P* value
**Low**							
	Low	0 (reference)	—	0 (reference)	—	0 (reference)	—
	Intermediate	–1.27 (–2.05 to –0.49)	.001	–1.41 (–2.17 to –0.64)	<.001	–0.88 (–1.58 to –0.19)	.01
	High	–2.45 (–3.34 to –1.57)	<.001	–2.33 (–3.21 to –1.46)	<.001	–1.63 (–2.46 to –0.80)	<.001
**Intermediate**							
	Low	–2.07 (–2.93 to –1.22)	<.001	–2.03 (–2.87 to –1.19)	<.001	–1.35 (–2.13 to –0.58)	.001
	Intermediate	–2.12 (–2.89 to –1.35)	<.001	–2.06 (–2.82 to –1.30)	<.001	–1.23 (–1.93 to –0.53)	.001
	High	–2.67 (–3.51 to –1.83)	<.001	–2.67 (–3.50 to –1.83)	<.001	–1.81 (–2.58 to –1.05)	<.001
**High**							
	Low	–1.93 (–3.13 to –0.72)	.002	–1.58 (–2.76 to –0.40)	.009	–0.82 (–1.88 to 0.24)	.13
	Intermediate	–2.85 (–3.76 to –1.95)	<.001	–2.80 (–3.70 to –1.90)	<.001	–1.97 (–2.80 to –1.14)	<.001
	High	–3.55 (–4.39 to –2.71)	<.001	–3.35 (–4.18 to –2.53)	<.001	–2.08 ( –2.83 to –1.33)	<.001

^a^vPA: vigorous physical activity.

^b^mPA: moderate physical activity.

^c^Model A: unadjusted regression model.

^d^Model B: regression model adjusted for sociodemographic variables (sex, BMI, income).

^e^Model C: regression model adjusted for sociodemographic and behavioral variables (model B plus fruit and vegetable intake, alcohol use, and sleep quality).

## Discussion

### Principal Findings

This study examined the associations of overall PA, ST, and activity intensity with perceived stress among university students. In line with previous studies, female students report higher levels of perceived stress compared with male students. As hypothesized, perceived stress is inversely related to the amount of PA and positively associated with ST. Our results further indicate that the combined associations of PA and ST with perceived stress are stronger than compared with either alone. However, contrary to our last hypothesis, engaging in vPA did not reveal stronger associations with perceived stress compared with engaging in mPA.

### Associations of Physical Activity, Sedentary Time, and Perceived Stress

In our first hypothesis, we assumed that self-reported PA and ST are independently associated with perceived stress. For total PA, we were able to demonstrate a dose-response relationship with perceived stress, confirming our hypothesis that a higher level of overall PA is inversely associated with perceived stress, which is in line with previous studies among university students [27,52,53]. Several reasons may explain these associations from different perspectives. Presumably, there is no single mechanism that can explain this relationship on its own. Beneficial effects of PA on stress have been demonstrated including physiological mechanisms (eg, increased endorphin levels, body temperature, neurotransmitter secretion), cognitive behavioral effects (eg, distraction from negative feelings, mastery, self-efficacy), and inflammatory mechanisms (eg, cytokine release, reduction of visceral fat mass, increase in vagal tone) [23,54].

According to our categorization into low, intermediate, and high ST, which was based on previous epidemiological studies [51,55], our findings generally support preceding research indicating that ST among university students is positively associated with perceived stress [28,35,36]. However, our findings do not support the notion of a clear dose-response relationship. Though students in the high ST category reported the highest stress levels, students in the low or intermediate ST category revealed only negligible differences in perceived stress. This is partly in line with the only study among university students we are aware of examining the association between self-reported ST and perceived stress [28]. In their study, Felez-Nobrega et al [28] demonstrated that ST was significantly related to perceived stress on weekends and during leisure time on weekdays but not associated with total ST on weekdays. Unfortunately, we were not able to differentiate between domain-specific PA and ST, as evidence grows that contextual factors matter in the effect of PA and ST on mental health [28,56,57]. It is likely that the lack of a clear dose-response relationship between ST and perceived stress in our study might be ascribed to a too narrow categorization of ST level (<6 hours, 6 to 8 hours, >8 hours) and/or the lack of considering domain-specific ST.

### Combined Associations of Physical Activity and Sedentary Time With Perceived Stress

These results confirm our second hypothesis that the combined associations of PA and ST with perceived stress are stronger compared with either PA or ST alone. Acknowledging that studies on combined effects of PA and ST among university students are rare, this constitutes a starting point for future research. However, in line with a recent study among college students [36], the odds for experiencing increased perceived stress were lowest when students engaged in high levels of PA and low levels of ST. Similarly, the combination of insufficient PA and smartphone use, a correlate of ST, revealed stronger associations with high levels of perceived stress compared with only one or none of these health risk behaviors [58]. These current findings further support the notion of a dose-response relationship between the combinations of PA and ST and perceived stress. The results indicate that reducing ST and increasing PA both seem promising approaches for university mental health promotion programs. These scenarios could find their rationale in the observation that university students engage in high volumes of ST due to activities like attending lectures and studying that potentially place them at detrimental physical [49] and mental health states [59,60].

### Associations of Physical Activity Intensity and Perceived Stress

In our third hypothesis, we supposed that the association between PA and perceived stress was stronger for engaging in vPA compared with mPA. However, our results do not support this assumption. Intermediate compared with low engagement in mPA and vPA did not reveal different associations with perceived stress. However, the results indicate that high engagement in mPA is associated with marginally lower perceived stress compared with high engagement in vPA. Although this result does not challenge the finding that vPA has a stress protective potential among undergraduate students with high stress levels [52,61], it contradicts results from previous studies indicating that vPA is associated with mental health benefits beyond mPA [37]. In their study, Gerber et al [62] analyzed a convenience sample of 42 university students but implemented device-based PA assessments, representing a strength of their study, given that self-reports tend to overestimate PA due to social desirability and recall bias. Thus, we assume that the dissimilar assessment methods and a more representative sample in our study might have impacted the divergent results. Although vPA certainly plays an important role in the association of PA and stress, very strenuous exercises like high-intensity interval training might induce adverse effects. Paolucci et al [40] argue that physiological responses to high-intensity exercise might exacerbate physiological responses to psychological stressors and increase perceptions of stress. This would suggest a U-shaped dose-response relationship for PA and stress with an upper activity intensity limit and mPA as the optimal activity intensity for beneficial effects on perceived stress among university students.

### Interpretations and Generalizability

In general, findings of this study indicate that higher overall PA, lower ST, and high engagement in mPA and vPA are favorably associated with perceived stress among university students. These results corroborate previous research demonstrating that PA-related health behaviors, including higher levels of mPA, vPA, and low ST, are associated with the lowest levels of stress, depression, and anxiety [28,63,64].

University students, particularly females, are highly vulnerable to higher levels of stress, depressive symptoms, and anxiety and poorer overall mental health than the general population [15,65-67]. This can be attributed to reasons like the transition into adulthood and major lifestyle changes related to their studies [5,14,15]. Given that impaired mental health predicts study dropouts, there is a clear need for mental health promotion measures, particularly among female students. University students also tend to be at risk for unhealthy behaviors [22], including insufficient PA and prolonged ST [35]. These may as well be related to the university setting that inevitably promotes sedentary behavior during lectures, while studying, or even during socializing. It is essential to identify effective ways to cope with stress in the university setting. Engaging in PA is one of the most popular and common ways to manage stress, and the findings of this study strengthen this hypothesis. Lifestyle habits that develop during university studies are likely to manifest and thus may affect future health [68]. This points to the necessity of implementing student health management including opportunities for reducing sedentary behavior and PA promotion on campus.

### Strengths, Limitations, and Implications for Future Research

This is one of the few studies examining independent and combined associations of PA, ST, and activity intensity with perceived stress among university students. We recruited a representative sample size, covering all faculties of the university, and saw a response rate of 10%. The outcome variables and their categorization represent internationally accepted standards, and we were able to address sociodemographic and behavioral confounders in our analyses. In addition, we adopted the CHERRIES checklist to improve reporting results of online surveys. However, the results must be interpreted considering the following limitations. First, participants were recruited from a single university in Germany, limiting the generalizability of the results. Second, the cross-sectional study design does not allow for causal inferences. Given that being physically active has beneficial effects on perceived stress through neurophysiological, psychosocial, and behavioral mechanisms, we do not neglect the notion that high levels of perceived stress might also impact ST and PA behavior, which suggests a bidirectional association. To examine cause-effect relationships, prospective experimental study designs must be considered. Using self-reports is inherently subjected to bias, predisposing to under- or overestimations of actual behavior. Although this study was careful in the selection of questionnaires, device-based assessments of PA using wearables, at least in subsamples, might be alternatives for future studies to provide more accurate outcomes and more detailed information. Last, given the scope of this large-scale health survey that covered many items on student health behavior, no differentiation of domain-specific PA or ST was feasible, but is encouraged for future studies to better derive tailored health-promoting measures in the university setting.

### Conclusions

These findings indicate that total PA, low ST, and activity intensity are favorably associated with perceived stress among university students. The total volume of PA and ST and a moderate PA intensity influence the association with perceived stress and should be considered when developing student health promotion programs. In a sedentary university setting, where students are prone to prolonged ST, appropriate measures should be taken to enable students to maintain sufficient levels of PA for optimal health and well-being. These could encompass a PA-friendly environment, education and measures aiming to promote PA (eg, taking stairs instead of elevators), active lecture breaks, and university sports offerings. Assuming a bidirectional association between PA, ST, and perceived stress, these measures should include stress management strategies to help students adhere to an active lifestyle in stressful periods as well.

## Appendix

Multimedia Appendix 1 Checklist for Reporting Results of Internet E-Surveys (CHERRIES).

## Abbreviations

AUDIT-C: Alcohol Use Disorders Identification Test for Consumption
CHERRIES: Checklist for Reporting Results of Internet E-Surveys
IPAQ-SF: International Physical Activity Questionnaire Short Form
MET: metabolic equivalent of task
mPA: moderate physical activity
PA: physical activity
PSS: Perceived Stress Scale
sPSQI: short-form Pittsburgh Sleep Quality Index
ST: sedentary time
vPA: vigorous physical activity
