# Supplementary material for: Independent and Combined Associations of Physical Activity, Sedentary Time, and Activity Intensities With Perceived Stress Among University Students: Internet-Based Cross-Sectional Study
Source: JMIR Public Health Surveill. 2020 Nov 11;6(4):e20119. doi: 10.2196/20119 (PMC7688394; doi:10.2196/20119)
Supplement: Multimedia Appendix 1 [file publichealth_v6i4e20119_app1.pdf]

CHERRIES Statement—Checklist for Reporting Results of Internet E-Surveys

Item Category	Checklist Item	Explanation
**Design**		
	Describe survey design	In Page 5, under Methods →Study Design and Setting:<br>"…A cross-sectional online study has been implemented at the University of Münster in Germany…"
**IRB (Institutional Review Board) approval and informed consent process**		
	IRB approval	In Page 5, under Methods →Study Design and Setting:<br>"…Ethical approval has been obtained from the Institutional Review Board…"
	Informed consent	In Page 5, under Methods →Study Design and Setting:<br>"…Prior to the online survey, all participants gave informed consent…"
	Data protection	In Page 5, under Methods →Study Design and Setting:<br>"…The email included information on the length of completing the survey (20-30 minutes, 172 items on 15 pages), voluntariness, anonymity, data protection, and incentives (e.g. VIP tickets for sporting events)…."
**Development and pre-testing**		
	Development and testing	In Page 5, under Methods →Study Design and Setting:<br>"…The survey was carried out standardized (i.e. without randomization of items) using the evaluation software EvaSys version 8.0 with adaptive questioning (Electric Paper Evaluationssysteme GmbH, Lueneburg, Germany)…"

**Recruitment process and description of the sample having access to the questionnaire**		
	Open survey vs closed survey	In Page 5, under Methods →Study Design and Setting:<br>Closed survey, with "Students received an invitation email and were provided with an individual transaction number (TAN)."
	Contact mode	In Page 5, under Methods →Study Design and Setting:<br>"…The resulting 42,630 students were invited by e-mail to take part…"
	Advertising the survey	In Page 5, under Methods →Study Design and Setting:<br>"…The resulting 42,630 students were invited by e-mail to take part…"
**Survey administration**		
	Web/E-mail	In Page 5, under Methods →Study Design and Setting:<br>"…The resulting 42,630 students were invited by e-mail to take part… The survey was carried out standardized (i.e. without randomization of items) using the evaluation software EvaSys version 8.0 with adaptive questioning…"<br><br>Invitation via email providing a link to a website with the online survey (EvaSys), where responses were automatically captured.
	Context	In Page 5, under Methods →Study Design and Setting
	Mandatory / voluntary	In Page 5, under Methods →Study Design and Setting:<br>Voluntary, "…The email included information on the length of completing the survey…, voluntariness, anonymity, data protection…"

	Incentives	In Page 5, under Methods →Study Design and Setting:<br>"…Students received an invitation email and were provided with an individual transaction number (TAN). The email included information on the length of completing the survey (20-30 minutes, 172 items on 15 pages), voluntariness, anonymity, data protection, and incentives (e.g. VIP tickets for sporting events)."
	Time/Date	In Page 5, under Methods →Study Design and Setting:<br>"…The health survey was carried out during the summer term 2019…"
	Randomization of items or questionnaires	In Page 5, under Methods →Study Design and Setting:<br>"The survey was carried out standardized (i.e. without randomization of items) using the evaluation software EvaSys version 8.0 …"
	Adaptive questioning	In Page 5, under Methods →Study Design and Setting:<br>"The survey was carried out standardized (i.e. without randomization of items) using the evaluation software EvaSys version 8.0 with adaptive questioning…"
	Number of items	In Page 5, under Methods →Study Design and Setting:<br>"…The email included information on the length of completing the survey (20-30 minutes, 172 items on 15 pages) …"
	Number of screens (pages)	In Page 5, under Methods →Study Design and Setting:<br>"…The email included information on the length of completing the survey (20-30 minutes, 172 items on 15 pages) …"
	Completeness check	In Page 5, under Methods →Study Design and Setting:<br>"…No completeness check was available. However, incomplete surveys were captured as well…"
	Review step	In Page 5, under Methods →Study Design and Setting: YES

**Response rates**		
	Unique site visitor	In Page 5, under Methods →Study Design and Setting:<br>"…Students received an invitation email and were provided with an individual transaction number (TAN)…"
	View rate (ratio of unique survey visitors/unique site visitors)	Not applicable
	Participation rate (ratio of unique visitors who agreed to participate/unique first survey page visitors)	In Page 7, under Results →Participants and Descriptive Data:<br>"A total of 4,189 students participated in this online survey, resulting in an overall response rate of 10% (range 7.2% to 22.1% among the 21 university departments)…"
	Completion rate (ratio of users who finished the survey/users who agreed to participate)	In Page 7, under Results →Participants and Descriptive Data:<br>"A total of 4,189 students participated in this online survey, resulting in an overall response rate of 10% (range 7.2% to 22.1% among the 21 university departments)…"
**Preventing multiple entries from the same individual**		
	Cookies used	Not applicable
	IP check	Not applicable
	Log life analysis	Not applicable

	Registration	In Page 5, under Methods →Study Design and Setting:<br>"…Students received an invitation email and were provided with an individual transaction number (TAN)…"
**Analysis**		
	Handling of incomplete questionnaires	In Page 5, under Measurements → Physical Activity:<br>For example in Physical Activity, "…Missing PA or ST data were considered as completely missing for this case and not considered for statistical analysis (n=80)…"
	Questionnaires submitted with an atypical timestamp	Not applicable
	Statistical correction	In Page 6, under Measurements → Perceived Stress:<br>"…Ipsative mean imputation was used (n=70) when not more than one item of the complete scale was missing. In case of two or more missing items, the subject was not considered for statistical analyses (n=14)…"
